# Epigenetic Modulation of miR-122 Facilitates Human Embryonic Stem Cell Self-Renewal and Hepatocellular Carcinoma Proliferation

**DOI:** 10.1371/journal.pone.0027740

**Published:** 2011-11-28

**Authors:** Christine J. Jung, Sushma Iyengar, Kimberly R. Blahnik, Tijess P. Ajuha, Joy X. Jiang, Peggy J. Farnham, Mark Zern

**Affiliations:** 1 Transplant Research Program, University of California Davis Medical Center, Sacramento, California, United States of America; 2 Department of Pharmacology, The Genome Center, University of California Davis, Davis, California, United States of America; 3 Department of Internal Medicine, Division of Gastroenterology and Hepatology, University of California Davis Medical Center, Sacramento, California, United States of America; Ohio State University Medical Center, United States of America

## Abstract

The self-renewal capacity ascribed to hESCs is paralleled in cancer cell proliferation, suggesting that a common network of genes may facilitate the promotion of these traits. However, the molecular mechanisms that are involved in regulating the silencing of these genes as stem cells differentiate into quiescent cellular lineages remain poorly understood. Here, we show that a differentiated cell specific miR-122 exemplifies this regulatory attribute by suppressing the translation of a gene, *Pkm2*, which is commonly enriched in hESCs and liver cancer cells (HCCs), and facilitates self-renewal and proliferation. Through a series of gene expression analysis, we show that miR-122 expression is highly elevated in quiescent human primary hepatocytes (hPHs) but lost or attenuated in hESCs and HCCs, while an opposing expression pattern is observed for *Pkm2*. Depleting hESCs and HCCs of *Pkm2*, or overexpressing miR-122, leads to a common deficiency in self-renewal and proliferation. Likewise, during the differentiation process of hESCs into hepatocytes, a reciprocal expression pattern is observed between miR-122 and *Pkm2*. An examination of the genomic region upstream of miR-122 uncovered hyper-methylation in hESCs and HCCs, while the same region is de-methylated and occupied by a transcription initiating protein, RNA polymerase II (RNAPII), in hPHs. These findings indicate that one possible mechanism by which hESC self-renewal is modulated in quiescent hepatic derivatives of hESCs is through the regulatory activity of a differentiated cell-specific miR-122, and that a failure to properly turn “on” this miRNA is observed in uncontrollably proliferating HCCs.

## Introduction

One of the hallmark traits ascribed to human embryonic stem cells (hESCs) is a capacity for extensive self-renewal [1]. While the indefinite cell division capacity of hESCs is an asset as an unlimited source of pluripotent cells for use in tissue replacement, the apparent similarity between stem cell self-renewal and cancer cell proliferation has raised concerns over the tumorigenic risk of hESCs and their derivatives in the transplanted tissue [2], [3]. The phenotypic parallels between these two classes of cells have raised speculation that a common network of genes may be involved in facilitating the promotion of these traits, and that a failure to properly control the silencing of these genes in quiescent derivatives of hESCs may give rise to uncontrolled proliferation that is typical of many cancer cells. However, to date, the molecular mechanisms that involved in governing the regulation of this shared network of genes in hESCs and cancer cells remain poorly understood.

Although both hESCs and cancer cells possess an extensive cell division capacity that gives rise to identical progenies, unlike cancer cell proliferation, stem cell self-renewal is tightly regulated by extrinsic signals and intrinsic factors in the developing embryo [4]–[7]. During development, the transition from pluripotent to lineage specified cells is carefully orchestrated by turning on genes that are required for the specification of lineage determining cells and restricting the expression of genes that promote self-renewal [8], [9]. As stem cells move further along the differentiation pathway toward various cell fate lineages, many differentiated cell types enter a quiescent or terminally differentiated state by exiting the cell cycle. This is especially evident during liver morphogenesis when stem cells differentiate into quiescent hepatocytes. While much attention has been focused on elucidating the mechanisms that are involved in promoting ESC self-renewal, less attention has been given to understanding how this trait is down-regulated as hESCs differentiate into quiescent cell types, and whether a failure to properly turn “on” these regulatory mechanisms can be observed in proliferating cancer cells.

In this study, we have examined the role of a differentiated cell-specific miR-122 as a regulator of a common network of genes that promote the facilitation of stem cell self-renewal as well as hepatocellular carcinoma cell (HCC) proliferation. microRNAs (miRNAs) are endogenous regulatory RNAs that refine and limit the expression of mRNAs post transcription by suppressing translation, and influencing the ultimate proteome [10]. Even though the number of miRNAs in the human genome is small relative to the number of protein coding genes, this family of RNAs has been described as one of the master regulators of the cell, because of their involvement in a wide range of cellular or organ programs including, for example, maintaining stemness, guiding differentiation, and functioning as tumor suppressors or oncomirs depending on the cell type in which they are expressed [11]–[15]. In the liver, miR-122 has been described as a critical facilitator of various homeostatic functions, notably, fatty acid and cholesterol metabolism, as well as hepatitis C viral replication [16]–[20]. Of relevance to this study are recent reports demonstrating the role of miR-122 as a potential tumor suppressor, because of its ability to regulate cell cycle progression and metastasis in liver cancer cells [21]–[27]. The ability of miR-122 to inhibit cell cycle progression in liver cancer cells raises the possibility that this miRNAs may also regulate hESC cell self-renewal during hepatocyte differentiation, by suppressing a common network of genes that promote hESC self-renewal as well as HCC proliferation.

Here, we show that miR-122 is highly enriched in differentiated human primary hepatocytes (hPHs), and functions as a modulator of hESCs self-renewal and HCC proliferation by suppressing the translation of a metabolic protein, PKM2. Through a series of global gene expression analysis, we show that miR-122 expression is either lost or severely attenuated in hESCs and HCCs, while an opposing expression pattern is observed for *Pkm2*. Both depleting hESCs and HCCs of *Pkm2*, or gain of miR-122 function, leads to a notable deficiency in self-renewal and proliferation. Likewise, during the differentiation process of hESCs into hepatocytes, a reciprocal expression pattern is observed between miR-122 and *Pkm2*, suggesting a possible role for this miRNA as a modulator of self-renewal during hepatic lineage specification. An examination of the genomic region up-steam of miR-122 uncovered hyper-methylation in hESCs and HCCs, while the same region is de-methylated and occupied by a transcription initiating protein, RNA polymerase II (RNAPII), in hPHs. Our findings suggest that one possible mechanism by which hESC self-renewal and HCC proliferation are modulated is through the regulatory activity of a differentiated cell-specific miR-122, which directly suppresses the translation of a gene, *Pkm2*, that is commonly enriched in hESCs and HCCs, and plays a role as a facilitator of these traits.

## Results

### mRNAs that are commonly enriched in hESCs and HCCs possess Gene Ontology (GO) functional terms describing actively dividing cells

**Figure 1:**
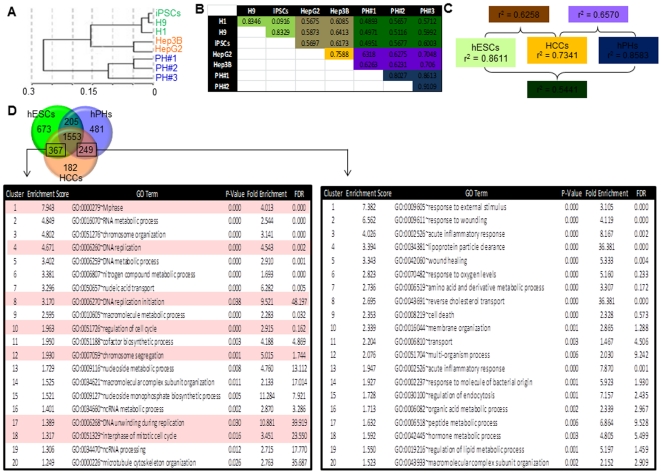
mRNA enrichment patterns and GO analysis in hESCs, HCCs and hPHs. (A) The cluster dendogram shows the similarity of mRNA expression patterns within each cell class, while marked differences exist among stem cells (green), cancer cells (red) and differentiated hepatocytes (blue). The scale on the X axis displays the level of differences among the samples based on Pearson correlation using a 1-r distance measure. (B) Pearson correlation computation of all the samples. Coefficient values within the same color scheme distinguishes the classes of cells that were correlated: ESCs vs. ESCs (green), ESCs vs. HCCs (brown), ESCs vs. hPHs (dark green), HCCs vs. HCCs (orange), HCCs vs. hPHs (purple) and hPHs vs. hPHs (blue). (C) Average correlation values from each color category from Figure 1B. (D) The data generated from the mRNA expression arrays were normalized, log_2_-transformed, categorized as “enriched” based on an enrichment parameter set at log_2_ value of 7.0 or higher. The enriched genes are distributed according to the Venn diagram. The three circles represent hESCs (green), hPHs (blue), and HCCs (orange). GO functional annotation clusters were derived from mRNAs that were commonly enriched in HCCs and hESCs (367), and HCCs and hPHs (249). The clusters were ranked according to the EASE scores of each term, and the top 20 clusters are listed in the tables. For each cluster, the GO term with the lowest P-value is shown as a representative functional term. The columns list the cluster ID, the enrichment score, GO ID of each term, P-value, fold enrichment, and the false discovery rate (FDR) of each term. Boxed in red are GO terms describing cell cycle regulation.

As a starting point of our study, we sought to determine whether the similarity between hESC self-renewal and HCC proliferation is reflected in the functional properties of mRNAs that are commonly enriched in these classes of cells. To do this, we performed a series of global mRNA expression profiles in hESCs, hPHs and HCCs (HepG2 and Hep3B), and compared their expression patterns. We show using hierchical clustering and Pearson correlation analyses that global gene expression patterns within each cell class are highly similar, while notable differences are observed among the three classes of cells (Figure 1A–C). These observations indicate that hESCs, hPHs and HCCs possess gene expression signatures that are specific and unique for each class of cells. Pearson correlation further revealed that while a large dissimilarity exists between the correlation coefficients undifferentiated hESCs and differentiated hPHs (mean r^2^ = 0.5441), HCCs appear to possess a signature that resembles ESCs (mean r^2^ = 0.6258) as well as hPHs (mean r^2^ = 0.6570), relative to the correlation between hPHs and hESCs (Figures 1B and 1C). These observations led us to speculate whether a large fraction of genes that are commonly enriched in HCCs and hPHs may reflect a common tissue origin, while genes that are commonly enriched in HCCs and hESCs may possess functional relevance to self-renewal and proliferation. In order to evaluate this, the normalized data generated from the mRNA arrays were log_2_-transformed, and categorized as “enriched” based on a parameter set at log_2_ value of 7.0 or higher. For HCCs, genes were considered “enriched” if both HepG2 and Hep3B cells met the enrichment criteria. We found that among the 17,172 mRNAs that were evaluated, 367 (2.14%) were commonly enriched in hESCs and HCCs, but not in hPHs, and 249 (1.45%) were commonly enriched in hPHs and HCCs, but not in hESCs (Figure 1D). To evaluate the functional properties of these sets of genes, the program DAVID [28] was utilized to assess the functional classification of each gene based on Gene Ontology (GO) descriptions. GO classifications were then evaluated further using a feature in DAVID which permits the clustering these genes according to the similarity of their functional terms. Not surprisingly, we found that a large fraction of the gene set that is commonly enriched in hESCs and HCCs generated GO clusters describing functional activities that are highly relevant for actively dividing cells (e.g., M phase, DNA replication, DNA replication initiation, regulation of cell cycle, and chromosome segregation) (Figure 1D, highlighted in red). In contrast, mRNAs commonly enriched in HCCs and hPHs, but not in hESCs, generated generated clusters describing functions that are generally associated with the homeostasis of differentiated cells (e.g., response to external stimulus, response to wounding, and immune and inflammatory responses), and various functions relating to the liver (e.g., reverse cholesterol transport and lipid metabolic process) (Figure 1D). Taken together, these results show that a large fraction of mRNAs that are commonly up-regulated in both hESCs and HCCs, but not in hPHs, embody GO functional descriptions that reflect the self-renewal and proliferation traits ascribed to stem cells and cancer cells.

### Differentiated cell-specific miR-122 is highly expressed in hPHs, and predicted to target a hepatic progenitor stem cell associated gene, *Pkm2*, that is commonly enriched in hESCs and HCCs

**Figure 2:**
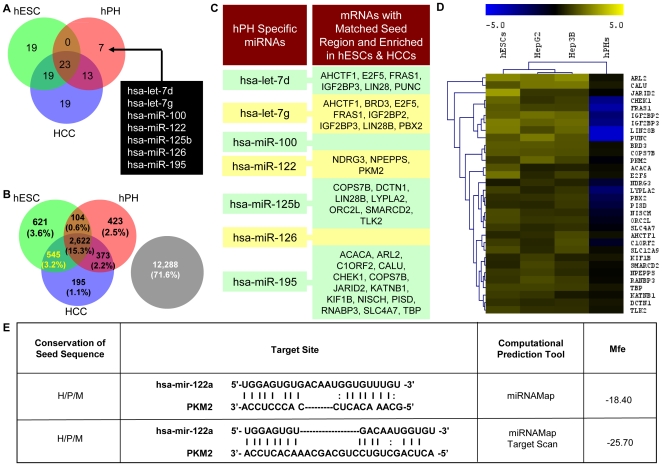
miRNA enrichment pattern and the predicted target mRNAs. (A) The data generated from the miRNA arrays were normalized, log_2_-transformed, categorized as “enriched” based on an enrichment parameter set at log_2_ value of 6.0 or higher, and distributed according to the Venn diagram. For HCCs, genes were considered enriched if both HepG2 and Hep3B cells met the enrichment criteria. The three circles in the Venn diagram represent the number of enriched gene sets in hESCs (green), hPHs (red), and HCCs (blue). Based on this criteria, seven miRNAs were identified to be exclusively enriched in hPHs. (B) mRNA enrichment pattern as shown in Figure 1. (C) miRNAs that were exclusively enriched in hPHs are listed in the first column. The second column lists 18 mRNAs that were commonly enriched in hESCs and HCCs, and predicted to be targeted by hPH-specific miRNAs (Figure 2A) based on both Target Scan and miRNAMap 2.0. (D) Enrichment pattern and hierarchical clustering of mRNAs that are predicted to be targeted by hPH-specific miRNAs from Figure 2C. Gene symbols are listed along the right column of the heatmap. The scale bar across the top depicts standard deviation change from the mean. (E) Predicted duplex formation between miR-122 and *Pkm2* 3′UTR based on miRNAMap and Target Scan. The lower Mfe values reflect the energetically more probable hybridizations between the miRNAs and the target genes. The seed sequences are conserved between *Homo sapiens* (H), *Pan paniscus* (P) and *Mus musculus* (M). Additional conservation information and the genomic locations of miR-122 and the 3′UTR of *Pkm2* are shown in Figures S1 and S2.

Because mRNAs commonly up-regulated in hESCs and HCCs embody GO functional descriptions associated with self-renewal and proliferation, we sought to determine whether a differentiated cell-specific miR-122 facilitates the suppression of these traits in hESCs and HCCs by targeting mRNAs commonly up-regulated in these two classes of cells. To do this, we performed global miRNA expression profiles on hESCs, HCCs and hPHs to determine whether miR-122 is highly expressed in hPHs, but attenuated in hESCs and HCCs. By using this approach, we identified seven miRNAs, including miR-122, that are exclusively enriched in hPHs (Figure 2A). We then utilized miRNA target prediction tools provided by miRNAMap 2.0 [29] and Target Scan [30] to examine whether these seven miRNAs contain seed sequences that are predicted to target the 3′UTR of the 367 mRNAs that are commonly enriched in hESCs and HCCs (Figure 1D and Figure 2B). Our analysis uncovered 18 out of 367 mRNAs that are predicted to be targeted by at least one of the seven hPH-specific miRNAs (Figure 2C and 2D). In this list, we observed that miR-122 is predicted to target three genes (*Ndrg3*, *Npepps*, *Pkm2*). Among this list of genes that are predicted to be targeted by miR-122, we selected miR-122:*Pkm2* pair for an in-depth analysis, because the embryonic isoform of pyruvate kinase (*Pkm2*) is reported in the literature to be highly expressed in actively proliferating hepatic progenitor stem cells (hepatoblasts), as well as many cancer cells [31]–[34]. An examination of the 3′UTR of *Pkm2* using Target Scan and miRNAMap 2.0 revealed two potential binding sites for miR-122 (Figure 2E). We, therefore, speculated that *Pkm2* may facilitate the promotion of hESC self-renewal and HCC proliferation, and that miR-122 may play a role as a modulator of these traits in differentiated hepatocytes by suppressing the translation of *Pkm2*.

### hPH-specific miR-122 suppresses the translation of *Pkm2*

**Figure 3:**
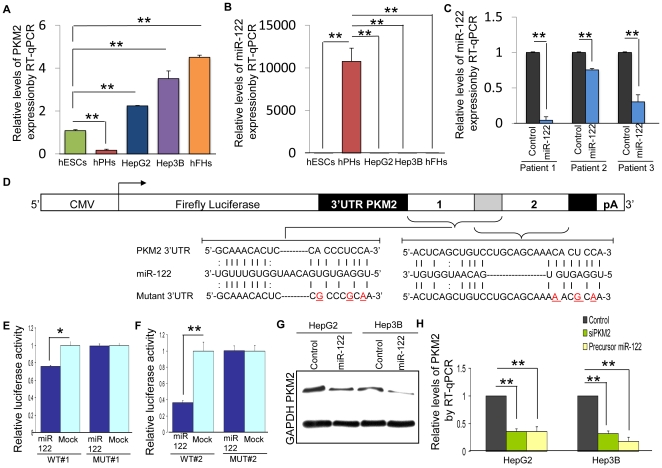
miR-122 hybridizes to the 3′UTR of *Pkm2* and induces endogenous translation suppression. (A) RT-qPCR of *Pkm2* expression in hESCs, hPHs, HepG2, Hep3B and hFHs. (B) RT-qPCR of miR-122 expression in hESCs, hPHs, HepG2, Hep3B and hFHs. (C) RT-qPCR of miR-122 expression in human liver tumor samples relative to the normal tissue counterparts. (D) Outline of the luciferase reporter construct for validating the interaction of miR-122 with the two overlapping target sites within the 3′UTR of *Pkm2*. miRNA response elements (MREs) that were predicted by miRNAMap (WT#1) (chr15:70,278,510–70,278,528) and both miRNAMap and Target Scan (WT#2) (chr15:70,278,518–70,278,545) as shown in Figure 2E were inserted into separate miR-REPORT vectors, immediately downstream of the *Firefly luciferase* gene, which is under the control of a constitutive CMV promoter. Downstream of MRE insert is polyadenylation signal (pA). The overlapping target sequences are colored in gray. In the mutant reporter constructs of WT#1 and WT#2 (MUT#1 and MUT#2), a three-basepair mismatch mutation (red, underlined) was introduced into the seed region. Reporter constructs as these are a standard method used to provide experimental evidence that miRNAs can directly repress translation initiation. (E, F) Each Firefly luciferase construct was cotransfected with precursor miR-122 molecules or mock miRNA molecules into HepG2 cells, and assayed for Firefly luciferase activity after 36 hours. Renilla luciferase construct was used as a normalization tool. The bar graphs show a decrease in luciferase activity when the cells were cotransfected with the wild-type (WT#1 or WT#2) MREs and precursor miR-122 molecules compared to the mock control. The difference in luciferase activity was not observed in mutant MREs (MUT#1 or MUT#2). (G) Detection of PKM2 by Western blot assay in HepG2 and Hep3B cells 3 days after transduction with lentivirus over-expressing miR-122 (miR-122) or scambled oligo sequence as control (C). (H) A decreased level of *Pkm2* transcripts was observed 36 hours after transfecting the HepG2 and Hep3B cells with either *siPkm2* or precursor miR-122 molecules compared to the control cells that were transfected with mock miRNA molecules. For all statistical analyses, n = 3; error bars represent s.e.m.; Student's t-test results are indicated by (*) P<0.05 and (**) P<0.01 relative to the control.

To determine whether miR-122 is capable of attenuates the expression of *Pkm2 in vitro*, we first validated the opposing expression patterns of *Pkm2* and miR-122 in hESCs, HCCs and hPHs, using quantitative real time PCR (RT-qPCR). In addition to these cell types, telomerase-immortalized human fetal hepatocytes (hFHs) were evaluated as a representative sample of hepatic progenitor stem cells – during liver morphogenesis, actively proliferating fetal hepatocytes give rise to quiescent mature hepatocytes. The RT-qPCR results were consistent with the mRNA expression array data, and showed an elevated *Pkm2* expression in hESC and HCCs, while in hPHs, *Pkm2* was severely attenuated (Figure 3A). An opposite expression pattern was observed for miR-122 in these cell types (Figure 3B). As expected, hFHs mirrored the *Pkm*2 and miR-122 expression patterns that were observed in hESCs and HCCs.

Because established cell lines cannot fully recapitulate clinical malignancy, we also examined the enrichment pattern of miR-122 in primary liver tissues from three patients. Consistent with the observations in HCCs, we found that miR-122 expression was significantly attenuated in human liver tumor tissues (Figure 3C). These results indicate that miR-122 expression is pervasively attenuated in self-renewing hESCs and proliferating liver cancer cells, and espouses an opposing expression pattern to *Pkm2*.

We then performed a series of tests using a luciferase reporter vector to evaluate whether miR-122 hybridizes to the *in silico* predicted sites in the 3′UTR of *Pkm2* and inhibit translation (Figure 2E). To do this, a portion of the 3′UTR of *Pkm2* containing either of the two predicted miR-122 target sequences, and two derivative sequences with three-mismatch mutations, were cloned into separate luciferase reporter vectors (Figure 3D). Each of these vectors was then co-transfected into HepG2 cells with precursor miR-122 molecules or mock precursor miR molecules. The cells were assayed for luciferase activity 36 hours post-transfection. We found that HepG2 cells that were transfected with either of the two wild-type luciferase constructs resulted in a significant reduction of the luciferase activity in the presence of precursor miR-122 relative to the control (Figures 3E and 3F). Suppression of the luciferase activity was not observed when precursor miR-122 was co-transfected into HepG2 cells with luciferase constructs that contained the three-base mismatch mutation. These data indicate that miR-122 directly binds to the two predicted target sites in the 3′UTR of *Pkm2* and suppresses translation. Protein immunoblot assay validated the direct translational suppression of *Pkm2* by miR-122 (Figure 3G).

To ascertain the endogenous translational suppression of *Pkm2* by miR-122, we performed a series of loss-of-function analyses by ectopically transfecting HepG2 and Hep3B cells with *siPkm2*, precursor miR-122 molecules, or mock precursor miR molecules. The transfected cell cultures were assayed 36 hours later for the level of *Pkm2* expression using RT-qPCR. Consistent with the luciferase reporter assays, over-expression of miR-122 in HepG2 and Hep3B cells significantly reduced the level of *Pkm2* transcripts relative to the mock miR transfections (Figure 3H), indicating that miR-122 hybridizes to the 3′UTR of *Pkm2* and induces transcript destabilization. These results suggest that an absence of miR-122 expression in hESCs and HCCs may permit an increased expression of *Pkm2*. In contrast, the high level of miR-122 expression in hPHs likely attenuates the endogenous *Pkm2* expression.

### *Pkm2* facilitates the promotion of hESC self-renewal and HCC proliferation

**Figure 4:**
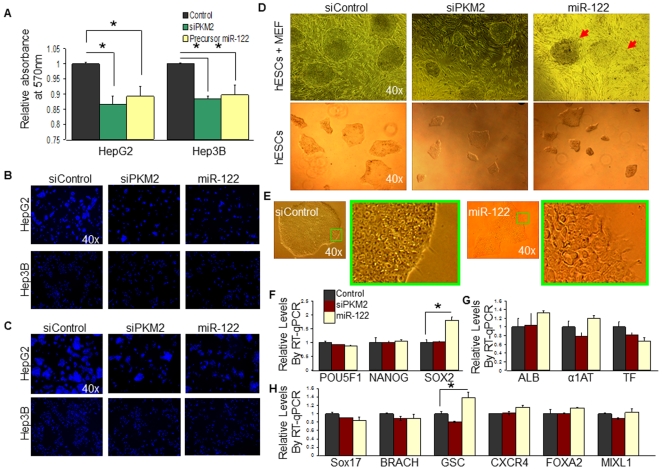
Reduction of endogenous *Pkm2* expression by miR-122 modulates cellular proliferation. (A) HepG2 and Hep3B cells were transfected with *siPkm2*, precursor miR-122 molecules, or mock miRNA molecules, and evaluated for cellular proliferation using an MTT based assay 36 hours post-transfection. n = 3; error bars represent s.e.m.; Student's t-test results are indicated by (*) P<0.05 and (**) P<0.01 relative to the control. (B,C) HepG2 and Hep3B cells were transfected with *siPkm2*, precursor miR-122 molecules, or mock miRNA molecules and stained with Hoecht 24 hours (4B) or 36 hours (4C) post-transfection. (D) Feeder dependent hESCs were transfected with *siPkm2*, precursor miR-122 molecules, or mock miRNA molecules. 36 hours post-transfection the morphology of the cells were observed. In general, smaller hESC colonies were observed when transfected with *siPkm2* compared to the control. hESCs that were transfected with miRNA-122 molecules showed more colonies with morphologically differentiated regions (shown by red arrows). (E) Feeder independent hESCs transfected with mock miRNA molecules showed tight cell arrangement, while hESCs transfected with miR-122 frequently showed lose and unhealthy cellular morphology. Green boxes show enlarged images. (F,G,H) RT-qPCR of feeder independent hESCs transfected with *siPkm2*, precursor miR-122 molecules, or mock miRNA molecules showed no significant change in *Pou5f1* and *Nanog* expression compared to the control (4F). A significant increase in *Sox2* expression was observed in hESCs that were transfected with precursor miR-122 molecules. No significant change in mature hepatocyte markers (*Alb*, *α1At* and *Tf*) was observed (4G). RT-qPCR of early hepatic lineage markers revealed a significant increase in GSC expression when hESCs were transfected with precursor miR-122 molecules (4H). n = 3; error bars represent s.e.m.; Student's t-test results are indicated by (*) P<0.05 and (**) P<0.01 relative to the control.

**Figure 5:**
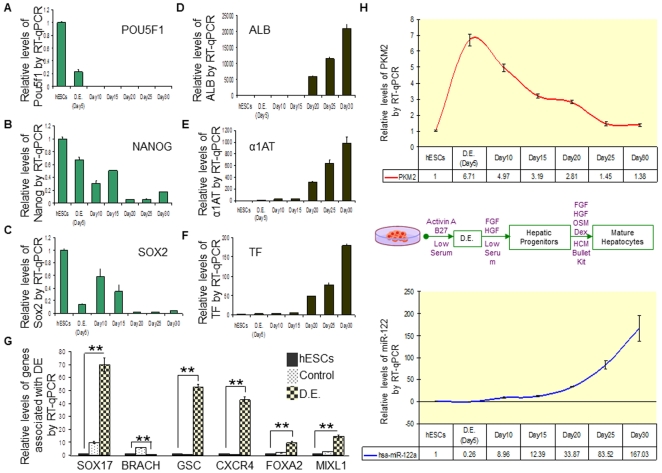
*Pkm2* and miR-122 expression patterns are inversely correlated during hepatic differentiation of hESCs. (A–C) RT-qPCR of hESC associated marker genes –*Pou5f1*, *Nanog* and *Sox2*– at 5 day intervals during the differentiation timeline, relative to day 0. n = 3; error bars represent s.e.m. Student's t-test results are indicated by (*) P<0.05 and (**) P<0.01 relative to hESCs (day 0). (D–F) RT-qPCR of mature hepatocyte marker genes –*Alb*, *α1At* and *Tf*– at 5 day intervals during the differentiation timeline, relative to day 0. n = 3, error bars represent s.e.m. Student's t-test results are indicated by (*) P<0.05 and (**) P<0.01 relative to hESCs (day 0). (G) RT-qRCR of genes reported to be up-regulated (*Cxcr4*, *Gsc*, *Sox17*, *Foxa2*, *Mixl1*) and down-regulated (*Brach*) in definitive endoderm. Solid bars represent the relative mRNA expression of hESCs. Dotted bars represent the relative mRNA expression of embryoid bodies (EBs) that were not treated with Activin A recombinant protein (Activin A induces definitive endoderm). Checkered bars represent the relative mRNA expression of EBs that were treated with Activin A recombinant protein to induce definitive endoderm cells. n = 3; error bars represent s.e.m. Student's t-test results are indicated by (*) P<0.05 and (**) P<0.01, relative to hESCs. (H) Relative levels of *Pkm2* expression compared to miR-122 expression via RT-qPCR at 5 day intervals during the differentiation timeline of hESCs into mature hepatocytes, relative to day 0 (undifferentiated hESCs). The diagram in between the two graphs describes the differentiation method corresponding to the differentiation timeline.

To determine whether *Pkm2* facilitates the promotion of hESC self-renewal and HCC proliferation, we performed a series of *in vitro* loss-of-function analysis by silencing the expression of *Pkm2* or over-expressing miR-122 in these cells. To do this, we transfected HepG2 and Hep3B cells with *siPkm2*, precursor miR-122 molecules, or mock control miR molecules, and evaluated the culture for cellular proliferation 36 hours following the transfection procedure using an MTT based assay. Compared to the control, cells that were treated with *siPkm2* or precursor miR-122 molecules yielded approximately 10–15% lower absorbance at 570 nm (Figure 4A). Hoechst stained images of HepG2 and Hep3B cells at 24 and 36 hours post-transfection showed a notable difference in the amount of nuclei between the control, and both the *siPkm2* and precursor miR-122 transfected conditions (Figures 4B and 4C). Quantification of the Hoechst stained nuclei from randomly chosen regions of the cultures validated the proliferation deficiency in the *siPkm2* and miR-122 transfected HCCs, relative to the control (Figures S3A and S3B). When hESCs were transfected with *siPkm2*, precursor miR-122 molecules, or mock control miR molecules, we observed, in general, a more frequent occurrence of smaller sized colonies in the *siPkm2* and miR-122 transfected conditions compared to the control (Figure 4D). In addition, the morphology of a larger fraction of hESC colonies in the miR-122 transfected condition appeared differentiated/unhealthy (Figure 4D and 4E). To examine the effect of knocking-down *Pkm2* and over-expressing miR-122 on hESC pluripotency, we assayed the expression patterns for hESC markers (*Pou5f1*, *Nanog*, *Sox2*) (Figure 5F), mature hepatocyte markers (*Alb*, *α1At*, *Tf*) (Figure 4G), and early differentiation markers following the transfection procedure (*Sox17*, *Brach*, *Gsc*, *Cxcr4*, *Foxa2*, *Mixl1*) (Figure 4H). We observed that hESCs that were transfected with *siPkm2* maintained similar expression levels of these genes relative to the control. In contrast, hESCs that were transfected with precursor miR-122 molecules revealed an increased expression of one of the stem cell markers (*Sox2*), as well as one of the early differentiation markers (*Gsc*). The markers for mature hepatocytes (*Alb*, *α1At*, *Tf*) did not show a statistically significant difference compared to the control when hESCs were transfected with *siPkm2* or miR-122 precursor molecules. These findings suggest that over-expressing miR-122 or knocking-down *Pkm2* in hESCs and HCCs modulates self-renewal and proliferation. However, while knocking-down *Pkm2* did not affect the ESC pluripotency associated genes that were observed, keeping the differentiated cell-specific miR-122 attenuated in hESCs appears essential in order to prevent the destabilization of a ESC marker genes, *Sox2*, and also, to inhibit the induction of an early differentiation gene, *Gsc*.

### An inverse expression pattern is observed between *Pkm2* and miR-122 during hepatocyte differentiation of hESCs

During the lineage specification process of hESCs into hepatocytes, the narrowing of the pluripotent potential is accompanied by a gradual loss of self-renewal. By the time stem cells have differentiated into mature hepatocytes, the cells are no longer self-renewing, and have settled into a quiescent state. Given the high enrichment of *Pkm2* in rapidly self-renewing hESCs and proliferating HCCs, and the role of miR-122 as a direct suppressor of this gene, we asked whether the loss of self-renewal during the hepatocyte differentiation process may be accompanied by a decreased *Pkm2* expression, and a corresponding increased expression of miR-122. To evaluate this, we utilized a three-step *in vitro* differentiation method for directing the specification of a large fraction of hESCs into hepatocyte-like cells [35]–[37], and examined the expression patterns of *Pkm2* and miR-122 at 5 day intervals during the differentiation timeline. To verify the efficacy of the differentiation method, we confirmed the gradual down-regulation of hESC associated genes (*Pou5f1*, *Nanog*, and *Sox2*) (Figures 5A–C), and the up-regulation of mature hepatocyte markers (*Alb*, *α1At*, and *Tf*) (Figures 5D–F) during the same 5 day intervals using RT-qPCR. In addition, we also confirmed the presence of definitive endoderm (DE) at day 5 of our differentiation timeline, which recapitulates the gastrulation process in the developing embryo, and gives rise to hepatic and pancreatic progenitor cells [37] (Figure 5G). Our results show that after day 5, the level of *Pkm2* expression began to decrease (Figure 5H). As stem cells moved further along the differentiation pathway, *Pkm2* expression continued to decrease with a corresponding increase in miR-122 expression. At day 30 of the differentiation timeline when a large fraction of hESCs have differentiated into mature haptocyte-like cells, a low level of *Pkm2* expression was observed. However, this level was still slightly higher than the level that was observed in hESCs. We suspect that at this may reflect a heterogeneous population of cells in the culture condition, composed of mature hepatocyte-like cells, as well as some remaining hepatic and other progenitor stem cells. Overall, these observations show that a reciprocal expression exists between *Pkm2* and miR-122 during hepatic lineage specification of hESCs. These findings suggest a role for miR-122 as a potential modulator of hESC self-renewal during the hepatocyte differentiation process by suppressing the translation of *Pkm2*.

### The up-stream genomic region of miR-122 is occupied by RNAPII in hPHs but hyper-methylated in hESCs and HCCs

**Figure 6:**
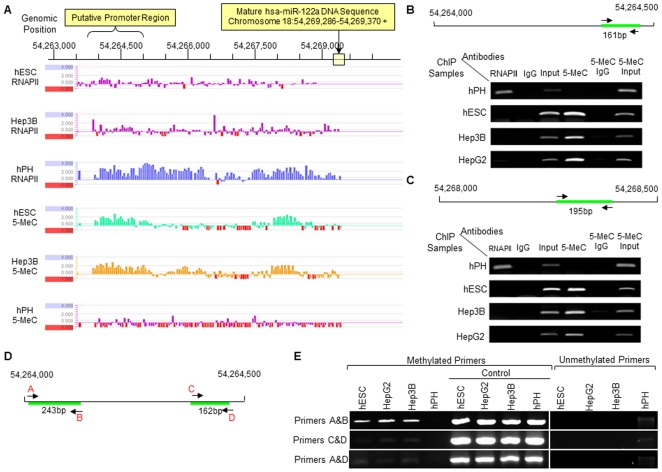
Methylation analysis of the genomic region upstream of miR-122 using ChIP-chip and bisulfite treatment. (A) High resolution map of RNAPII and 5-MeC binding sites on ChIP samples of hESCs, hPHs and Hep3B. The horizontal bar on the top of the figure indicates the genomic positions on chromosome 18, and the location of the mature miR-122 sequence in the genome is boxed in yellow. The predicted promoter sequence of miR-122 (Figure S4) is indicated above the horizontal bar with the genomic positions. ChIP-chip data are listed below the genomic position scale. Each vertical bar indicates the enrichment of a single probe as a log_2_ ratio value between the enriched ChIP sample and the input sample. (B,C) The genomic regions that appear in Figure 6A to be bound by RNAPII in hPHs and methylated in hESCs and HCCs (18:54,264,000–54,264,500 and 18:54,268,000–54,268,500) were verified by PCR. Arrows indicate the location of the primers, green bars indicate the amplified products, and the product sizes are listed below the green bars. (D) Diagram showing the predicted promoter region of miR-122 and primers used for amplification. The primers are indicated with letters A, B, C, D and colored in red. (E) PCR results showing that the methylation-specific primers amplifies the predicted promoter region (D) in hESCs and HCCs, while unmethylation-specific primers amplifies the same region in hPHs after bisulfite treatment. Control samples indicate hESCs, HCCs and hPHs that have not been bisulfite treated.

**Figure 7:**
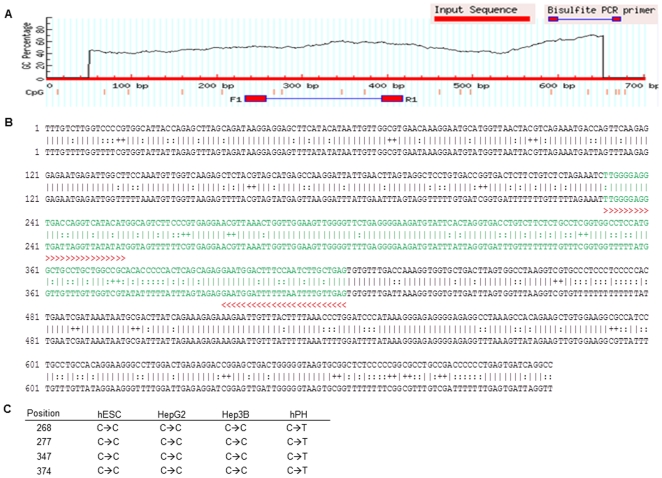
Bisulfite sequencing analysis of the predicted promoter region of miR-122 in hESCs, HCCs and hPHs. (A) Bisulfite sequencing PCR primer designed by the program MethPrimer using the predicted miR-122 promoter region as a template. (B) DNA sequence of the predicted promoter region of miR-122 entered into the MethPrimer program. The output shows the primers used to amplify the template with red colored arrows, the region amplified in green color, and the the cytocine (C) residue that would be converted into uracil if unmethylated is indicated with +. (C) The table shows the position of the cytocine from (B) that would be converted into uracil in absence of methylation in the bisulfite treated samples. Among the four cytocine positions, the bisulfite sequencing analysis showed that only the residues in hPHs were converted into uracil.

**Figure 8:**
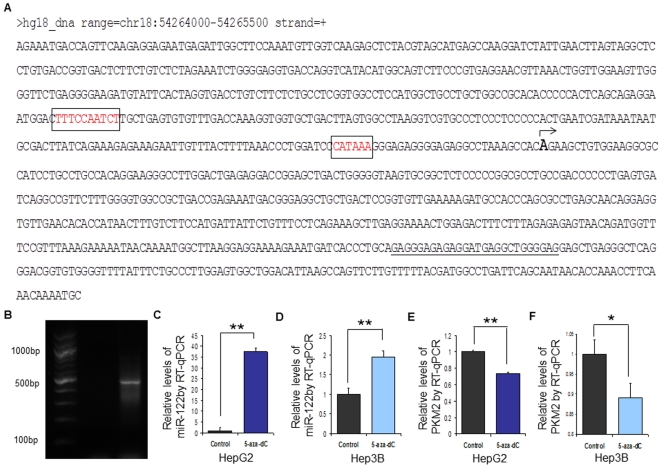
5′RACE promoter analysis of pri-miR-122. (A) The sequence shows the promoter region of pri-miR-122 as determined by 5′RACE. CAAT-box and TATA box are highlighted in red, and the TSS is indicated with an arrow. The primer used to amplify the 5′RACE product is underlined. The DNA sequence shows chr18:54,264,000–54,265,000 (NCBI36/hg18). (B) The primer used to determine the promoter region of pri-miR-122 using 5′RACE. (C,F) Relative expression of miR-122 after treating the HepG2 and Hep3B cells with 5-Aza-2′Deoxycytidine for 3 days. n = 3; error bars represent s.e.m. Student's t-test results are indicated by (*) P<0.05 and (**) P<0.01 relative to the control. (F,G) Relative expression of *Pkm2* after treating the HepG2 and Hep3B cells with 5-Aza-2′Deoxycytidine for 3 days. n = 3; error bars represent s.e.m. Student's t-test results are indicated by (*) P<0.05 and (**) P<0.01 relative to the untreated control. (H) Images of HepG2 and Hep3B cells 3 days after treatment with 5-Aza-2′-Deoxycytidine.

**Figure 9:**
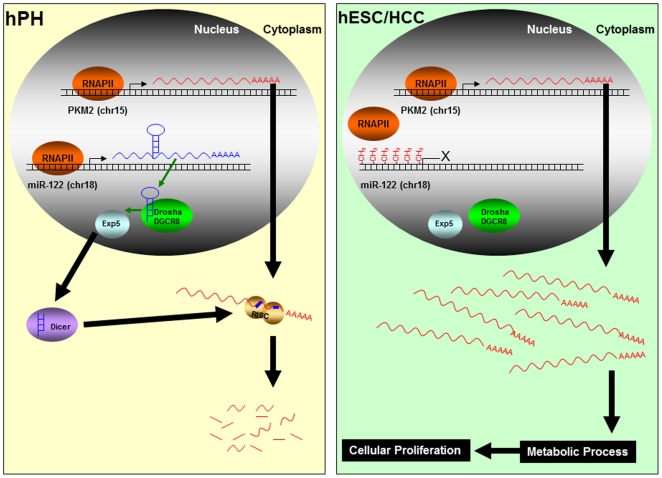
A model for miR-122 regulation of *Pkm2*. In hESCs and HCCs, hyper-methylation of the genomic region up-stream of miR-122 inhibits transcription. In hPHs, an absence of methylation allows the binding of RNAPII and initiation of miR-122 transcription.

In the preceding sections, we have shown that attenuating the expression of miR-122 in hESCs and HCCs allows for a constitutive expression of *Pkm2*, which in turn, facilitates the promotion of self-renewal and proliferation in these cells. We, therefore, sought to understand the molecular mechanism that may be involved in regulating the expression of this miRNAs in hESCs, HCCs and hPHs. To do this, we examined the 20 kb genomic region up-stream of miR-122, where the putative promoter sequence of this miRNA is likely to be found, for methylation status and binding activity of RNA Polymerase II (RNAPII) in hESCs, HCCs and hPHs. We chose to focus our attention on these epigenetic factors to because: 1) RNAPII has been described in the literature as the most prominent miRNA transcription initiating polymerase in metazoans [38]; and 2) hyper-methylation of the promoter region of genes has been reported to be frequently observed in aberrant gene silencing in a multitude of cancer cells [39]. We utilized a chromatin immunoprecipitation (ChIP) assay to examine the RNAPII binding activity and DNA methylation status of the 20 kb region upstream of the mature miR-122 sequences in the genomes of hESCs, hPHs and Hep3B. The ChIP data revealed that the upstream region of miR-122 in hPHs was covered with RNAPII, while in hESCs and Hep3B cells, the same region was hyper-methylated (Figure 6A). To verify these findings, we designed two PCR primer sets using templates from two separate regions (Chr18:54,264,000–54,265,000 and Chr18:54,268,000–54,269,000) that appeared to be hyper-methylated in hESCs and HCCs, and assayed for the presence of RNAPII and 5-MeC in chromatin immunoprecipitates of hESC, hPH and HCCs (Figures 6B and 6C). These experiments validated hypo-methylation and the presence of RNAPII in hPHs and hyper-methylation in HCCs and hESCs. To determine whether these regions contain a putative promoter sequence for miR-122, two programs, Promoter 2.0 [40] and BDGP [41], designed to identify possible promoter sequences in the genome were utilized. Both Promoter 2.0 and BDGP revealed a high likelihood that theregion between54,264,000–54,265,000 may contain a possible promoter sequence (Figure 6A; Figures S4A–C). We, therefore, performed additional experiments using bisulfite treatment of hESCs, HCCs and hPHs to validate whether this genomic region is hyper-methylated in hESCs and HCCs, while hypo- methylation in hPHs. By using PCR primers designed to amplify either the methylated or unmethylated regions of DNA after bisulfite treatment (Figure 6D), we show that the predicted promoter region of miR-122 is methylated in hESCs and HCCs, but unmethylated in hPHs (Figure 6E). We verified this finding using primers designed by the program MethPrimer program [42], which also showed that the predicted promoter region of miR-122 is unmethylated in hPHs, but methylated in hESCs and HCCs (Figures 7A–C). To confirm whether the promoter of miR-122 is located in this region, 5′RACE was performed to determine the transcription start site (TSS). By using this approach, we show that the TSS is located within the predicted promoter region of miR-122. In addition, the CCAAT-box and TATA-box are found at the upstream region of this TSS, which is frequently observed in the promoters many genes regulated by RNAPII (Figures 8A, B). Hence, the combined results of ChIP-chip, bisulfite sequencing, and 5′RACE analyses suggest that in hPHs, RNAPII binds to the hypo-methylated promoter of miR-122 and initiates transcription. In contrast, hyper-methylation of the same genomic region appears to prevent the binding of this transcription factor in hESCs and HCCs, andinhibits the transcription of this miRNA. In order to verify these findings *in vitro*, we treated the HepG2 and Hep3B cells with a demethylating reagent, 5-Aza-2′Deoxycytidine (5-aza-dC), and performed RT-qPCR 72 hours following the treatment to measure the level of miR-122 expression. We found that in HCCs that were treated with 5-aza-dC, a significant increase in miR-122 expression was observed compared to the control (Figures 8C and 8D). Moreover, we observed a decreased expression of *Pkm2* in the same conditions (Figures 8E and 8F). These findings suggest that miR-122 expression in hESCs, HCCs and hPHs may be associated with the methylation status and RNAPII binding activity at the promoter region of this gene (Figure 9).

## Discussion

The present work has provided evidence to suggest the involvement of a differentiated cell-specific miR-122 as a modulator of hESC self-renewal and HCC proliferation through a direct translational suppression of a gene, *Pkm2*, that is commonly enriched in these classes of cells. Both loss of *Pkm2* and gain of miR-122 function in hESCs and HCCs lead to a severe deficiency in self-renewal and proliferation, and during the differentiation process of hESCs into hepatocytes, a reciprocal expression pattern is observed between miR-122 and *Pkm2*. These findings suggest a possible role for miR-122 as a modulator of self-renewal during hepatic lineage specification, and that a failure to properly attenuate the expression of this miRNA is observed in proliferating HCCs. An examination of the genomic region up-steam of miR-122 uncovered hyper-methylation in hESCs and HCCs, while the same region is de-methylated and occupied by a transcription initiating protein, RNA polymerase II (RNAPII) in hPHs. Our findings suggest that hyper-methylation of the genomic region up-stream of miR-122 may interfere with the binding of RNAPII, which in turn, inhibits a proper initiation of miR-122 transcription (Figure 9). The coordinated interplay between miR-122 and *Pkm*2 suggests a novel and elegant mechanism for controlling the expression of a gene that may be beneficial for stem cells, but becomes undesirable as stem cells transition into a differentiated quiescent hepatic cell fate. By initiating the expression of miR-122, the differentiating cells of a hepatic lineage have evolved an effective method for reducing the cell of a transcript that they have outgrown, in order to pave a path to ushering in a new phenotype for which self-renewal is undesired. A failure to elevate the expression of miR-122 is observed in hepatocellular carcinoma cells, and its effect on cellular proliferation suggests that miR-122 embodies a function that is reminiscent of a tumor suppressor.

The activation of a specific metabolic pathway involving the enzyme pyruvate kinase (PK) in highly proliferating cancer cells has been described decades ago by Otto Warburg [43] – a metabolic process that is now known as aerobic glycolysis or the Warburg effect [44]. Since the initial discovery, various studies have shown that while *Pkm2* expression is primarily elevated in rapidly proliferating embryonic and progenitor stem cells, in terminally differentiated hepatocytes, PK is altered into a different isozyme, PK-L. Recent studies have demonstrated that in several cancer cells, the primary *Pkm2* transcript is re-expressed, however, in an altered isoform that is capable of interacting with a phosphor-tyrosine (pTyr) motif, which enables these cells to redirect oxidative phosphorylation towards aerobic glycolysis for growth and proliferation [5], [45], [46]. The ramifications of these findings are significant in reference to this study, as they suggest that in differentiated normal hepatocytes, miR-122 may function as a critical tumor suppressor by negatively regulating the level of *Pkm2* transcripts in the cell, thereby minimizing the likelihood that PKM2 may interact with pTyr. Hence, in addition to the many homeostatic functions that have been ascribed to miR-122 in the liver, this miRNA may also function as an early defense mechanism against a possible neoplastic transformation.

It remains to be seen whether silencing miR-122 in terminally differentiated hepatocytes may give rise to tumorigenesis. Such analysis is hampered at present by the lack of experimental strategy for keeping hPHs stable and/or alive for more than a few days after the isolation procedure from the cadaveric liver or hepatic resections, and the lack of techniques to identify, isolate, and culture mature hepatic derivatives from hESCs *in vitro*. Although several studies have knocked-down miR-122 expression in the mouse liver using ‘antagomirs,’ *Pkm2* up-regulation was not reported [19], [47]. For this reason, examining the relevance of miR-122 in stem and cancer cells of human origin helps elucidate the important tumor suppressor potential of this miRNA during the differentiation process of hESCs into quiescent hepatocytes. Such information may also provide a useful tool as a biomarker to evaluate the safety and efficacy of hepatic derivatives of hESCs in an *in vitro* differentiation culture system. Furthermore, studies like this would be helpful for exploring additional avenues through which to study liver cancer development, and facilitate the designing of early prognostic tools and treatment strategies for therapeutic intervention. Given the multifaceted role of miR-122 in the liver [16]–[27], it is likely that this miRNA suppresses genes other than *Pkm2* to modulate hESC self-renewal and HCC proliferation. For example, studies have shown that *Igf1r* is a direct target of miR-122, and may function as a mediator of HCC proliferation [48] (Figure S5). Future challenge will be to further elucidate the involvement of miR-122 as a regulator of hESC self-renewal and HCC proliferation through direct translational suppression of genes in addition to *Pkm2*, as well as the role of various other differentiated cell-specific miRNAs that have yet to be explored.

## Materials and Methods

### Cell culture and differentiation

#### Human ES cells

Human H9 ES cell line was obtained from the WiCell Research Institute (WA09), and maintained as a monolayer in 6-well (9.6 cm^2^) plates on gamma irradiated MEF feeder layers in Dulbecco's modified Eagle's medium/Ham's F-12 medium (Invitrogen) supplemented with 20% KnockOut Serum Replacement (Invitrogen), 4 ng/ml basic fibroblast growth factor (Invitrogen), 1 mM nonessential amino acids (Invitrogen), 2 mM L-Glutamine (Invitrogen), 100 u/ml penicillin/streptomycin (Invitrogen), and 0.55 mM 2-mercaptoethanol (Invitrogen). Cultures were passaged after collagenase treatment at a ratio of 1∶3 to 1∶6 every 4–6 days. Feeder-independent cultures of hESCs were maintained on matrigel (BD Biosciences) coated 6-well plates, in MEF conditioned medium. Induction of hESCs to definitive endoderm was initiated when hESC cultures were 75%–85% confluent, at which time they were washed with phosphate buffered saline (PBS) without calcium and magnesium (Invitrogen), and cultured in RPMI medium (Invitrogen) supplemented with 1XB27 (Invitrogen), 1 mM sodium butyrate (NaB) (Sigma Aldrich), 2 mM L-Glutamine, 100 u/ml penicilline/streptomycin, 0.5% KOSR, and 100 ng/ml Activin-A (R&D Systems). Five days later, differentiation of definitive endoderm into hepatic progenitor cell types was initiated by changing to MDBK-MM medium (Sigma-Aldrich), supplemented with 2 mM L-Glutamine, 100 u/ml penicilline/streptomycin, 10 ng/ml FGF-4, and 10 ng/ml HGF. Three days later, differentiation and enrichment of mature hepatocytes was initiated using the HCM Bullet Kit (Lonza). Human primary hepatocytes (hPHs) were obtained from the Liver Tissue Procurement and Distribution System at the University of Pittsburgh. Upon receiving the cells, they were washed three times in PBS without calcium and magnesium, and maintained in HCM Bullet Kit. Hepatocellular carcinoma cells HepG2 and Hep3B cells (American Type Culture Collection) were cultured per the manufacturer's instructions.

### mRNA and miRNA expression microarrays

#### mRNA arrays

RNA samples were isolated using the Qiagen RNeasy Kit (Qiagen). Prior to array experiments, the quality of the RNA samples were assayed using the Agilent Systems Bioanalyzer 2100. The total RNA from each sample was biotinylated and amplified for hybridization to Illumina Sentrix Expression Beadchip HumanRef-8 v3.0. This array platform consists of eight parallel strips, each strip composed of 24,500 probes from the NCBI refseq database (Build 36.2, Release 22). Arrays were processed and scanned per the manufacturer's instruction, and analyzed using the BeadStudio Software v3.0. All signals were normalized, log_2_-transformed, and ranked according to the log_2_ values. For each gene, the criterion for enrichment was set at a log_2_ value of 6.0 or higher. Hierarchical clustering was performed with average linkage and Pearson correlation. To generate the heatmap, values were centered and normalized to a mean of 0 and a standard deviation of 5. miRNA arrays miRNAs were prepared using the mirVana miRNA Isolation Kit from Ambion (Ambion), and purified and concentrated for small nucleic acids using flashPAGE Fractionator and flashPAGE Reaction Clean-up Kit (Ambion). Amine-modified nucleotides were added to the miRNAs in the samples, and labeled with Cy3 fluorescent dye per the manufacturer's instructions (Ambion). Labeled miRNAs were hybridized to Human miRNA Array v1.0 (Ambion) containing 365 human miRNA probes. Processed arrays were scanned at 10-um resolution using the GenePix 400B scanner (Axon Instruments). Raw data were normalized, log_2_-transformed, and ranked according to the log_2_ values. For each miRNA, the criteria for enrichment was set at log_2_ value of 6.0 or higher. All data is MIAME compliant, and the raw data has been deposited at GEO under accession number (GSE29907).

### DAVID analysis

Functional annotation clusterings were performed using the DAVID Bioinformatics Resources 2008. Gene sets that were common between HCCs and hESCs, and HCCs and hPHs, were subjected to separate clustering analyses. Each gene set was entered into DAVID's functional annotation clustering tool, which generated clusters of genes based on the similarity of the functional terms assigned to each gene. The clusters were then ranked according to the EASE scores of each term, and the top ten highest ranked clusters were selected for analysis. Within each cluster, the top five Gene Ontology terms with the lowest *P* values were selected as representative functional terms. If redundant terms were present (e.g., developmental process and cellular developmental process) the term with higher *P* value was eliminated.

### Chromatin immunoprecipitation assays and amplicon preparation

ChIP assays were performed as described at http://www.genomecenter.ucdavis.edu/farnham. Briefly, for 5-Methylcytidine (5-MeC) ChIPs, genomic DNA was extracted from 1×10^7^ cells of each sample, sonicated to an average size of 800 bp using a Bioruptor Sonicator (Diagenode), denatured, and incubated with the mouse monoclonal 5-MeC antibody (Eurogentec). RNAPolII ChIP was performed by cross-linking 1×10^7^ cells from each sample with 1.5% formaldehyde, after which time, nuclear extracts were prepared and chromatin was sonicated to an average size of 800 bp and incubated with mouse monoclonal RNA polymerase II antibody (Covance). For both 5-MeC and RNAPolII assays, secondary rabbit anti-mouse IgG (MP Biomedicals) was used, and the nonspecific rabbit IgG (Alpha Diagnostics) was used as a negative control. Immunoprecipitates were purified (QIAquick PCR purification kit, Qiagen), and amplicons were generated using the Sigma GenomePlex WGA2 Kit (Sigma-Aldrich). PCR positive and negative primers were used for ChIP samples and amplicons to confirm the validity of the final product.

### Assay and analysis of ChIP-chip data

The miR-122 promoter array was custom designed by tiling 20 kb region upstream from the mature miR-122 DNA sequence with 50-mer probes on 386K array platforms. Amplicons were labeled, hybridized to the promoter arrays, scanned, and analyzed for signal intensity of each probe by Roche-NimbleGen Systems, Inc. To confirm RNAPII binding and methylation, primers were designed from 501 bp regions of chr18:54,264,000–54,264,500 and chr18:54,268,000–54,268,500, and RT-PCR was performed using 1.0 ng of amplicons generated from RNAPII and 5-MeC ChIP samples (hESCs, hPHs, HepG2, and Hep3B).

### Quantitative RT-PCR

For mRNA RT-qPCR, total RNA was isolated using the Qiagen RNeasy Kit (Qiagen), and SuperScript III RT-qPCR Kit (Invitrogen) was used to synthesize cDNAs. For miRNA RT-qPCR, miRNAs were isolated using mirVana miRNA Isolation Kit (Ambion), and reverse transcribed into cDNAs using miRCURY LNA First Strand cDNA Kit (Exiqon). For both mRNAs and miRNAs, RT-qPCR mixture was prepared using ABI TaqMan or Sybr Master Mix (ABI), and RT-qPCR were performed on the ABI PRISM 7700 Sequence Detection System (ABI). The comparative 2−(ΔΔCt) method was used to determine the relative quantitative levels of mRNAs using GAPDH for mRNA normalization and Exiqon's Endogenous Control Primers (Exiqon) for miRNA normalization, and expressed in values as relative difference compared to the relevant controls.

### Luciferase reporter construct and assay

The luciferase reporter was constructed by cloning into pMiR-REPORT vector (Ambion) the target PKM2 sequences of miR-122 downstream of the firefly luciferase gene and verified by sequencing. The reporter plasmids were co-transfected with 20 pmol of precursor miR-122 molecules or mock precursor miRNA molecules (Ambion) into 50,000 HepG2 cells in 24-well plates using Lipofectamine 2000 (Invitrogen). Cells were lysed 36 hours later and processed for luciferase assay using the Luciferase Reporter Assay System (Promega).

### Lentiviral preparation and transduction

The production and titering of lentinvirus were carried out according to protocols from Tronolab (http://tronolab.epfl.ch).

### Western blot assay

Cells were lysed in RIPA buffer (Pierce). Twenty-five micrograms of cell lysates were analyzed on 12% SDS-PAGE. The antibodies used were PKM2 (Cell Signaling), IGF1R (Cell Signaling), GAPDH (Cell Signaling), anti-rabbit HRP-conjugated secondary antibody (Cell Signaling).

### Cell proliferation assay

50,000 HepG2 and Hep3B cells were grown on 24-well plates, and transfected with 20 pmol of precursor miR-122 molecules or mock precursor miRNA molecules (Ambion) using Lipofectamine 2000 (Invitrogen) 24 hours after initial plating of the cells. Cell proliferation was measured 24 hours later using the Cell Titer Cell Proliferation Assay (Promega) at 570 nm absorbance.

### 5′ rapid amplification of cDNA end (RACE) analysis

5′RACE was performed using the GeneRacer Kit (Invitrogen).

### Bisulfite analysis

hESCs, HCCs and hPHs were treated with CpGenome Bisulfite Modification Kit (Millipore). The genomic DNA was extracted and PCR amplified. The PCR products were cloned into plasmids for sequencing (Invitrogen).

### 5-Aza-2′-Deoxycytidine treatment

100,000 HepG2 and Hep3B cells were grown on 6-well plates and treated with 5 uM of 5-Aza-2′-Deoxycytidine for 3 days. Total RNA was extracted using the Qiagen RNeasy Kit (Qiagen), and cDNAs were synthesized using SuperScript III RT-qPCR Kit (Invitrogen).

## Supporting Information

Figure S1 **Genomic location and conservation of miR-122 and** ***Pkm2*** **.** (A) The location of miR-122 on chromosome 18 q arm is marked with a red bar. miR-122 is conserved in a large fraction of vertebrates. (B) The location of the *Pkm2* 3′UTR target sequence on chromosome 15 q arm is marked with a red bar. The target sequence predicted by MiRanda is located on 15:70,278,518–70,278,545 and the target sequence predicted by RNAhybrid is located on 15:70,278,510–70,278,528. *Pkm2* is conserved in a large fraction of vertebrates. Data and images were generated using the UCSC Genome Browser (genome.ucsc.edu).
(TIF)

Figure S2 **Genomic location and conservation of miR-122 and** ***Pkm2*** **.** (A) The location of *Pkm2* is indicated by a blue bar, and the predicted hybridization site by miR-122 is indicated by the purple bar. The target sequence is predicted by Target Scan computational tool. The large boxed region shows that the target sequence of miR-122 in the 3′UTR of *Pkm2* is conserved among a large number of species. Data and images were generated using the Target Scan computation prediction tool (www.targetscan.org).
(TIF)

Figure S3 **Reduction of endogenous** ***Pkm2*** **expression by miR-122 modulates cellular proliferation.** (A) HepG2 and Hep3B cells were transfected with siPkm2, precursor miR-122 molecules, or mock miRNA molecules. 24 hours (S1A) and 36 hours (S1B) post-transfection, five randomly chosen areas from 5 different wells in 24-well culture plates were stained with Hoecht and counted for the number of nuclei. n = 5; error bars represent s.e.m.; Student's t-test results are indicated by (*) P<0.05 and (**) P<0.01 relative to the control.
(TIF)

Figure S4 **Predicted transcription start site of vertebrate RNAPII using Promoter2.0 and BDGP.** (A) Genomic region between chr18:54,263,500–54,269,000 was evaluated for possible transcription start sequences using Promoter2.0 and Berkeley Drosophila Genome Project (BDGP). DNA sequence between chr18:54,263,800–54,265,000 yielded a highly likely promoter sequence (B) at position 800. (C) Predicted promoter sequences based on BDGP. Data and images were generated using Promoter 2.0 (cbs.dtu.dk) and BDGP (www.fruitfly.org).
(TIF)

Figure S5 **Overexpression of miR-122 is inversely correlated with IGF1R in HepG2 and Hep3B.** Western blot assay of IGF1R in HepG2 and Hep3B overexpressing miR-122.
(TIF)
