# Supplementary figures and images for: Epigenetic Modulation of miR-122 Facilitates Human Embryonic Stem Cell Self-Renewal and Hepatocellular Carcinoma Proliferation
Source: PLoS One. 2011 Nov 28;6(11):e27740. doi: 10.1371/journal.pone.0027740 (PMC3225380; doi:10.1371/journal.pone.0027740)

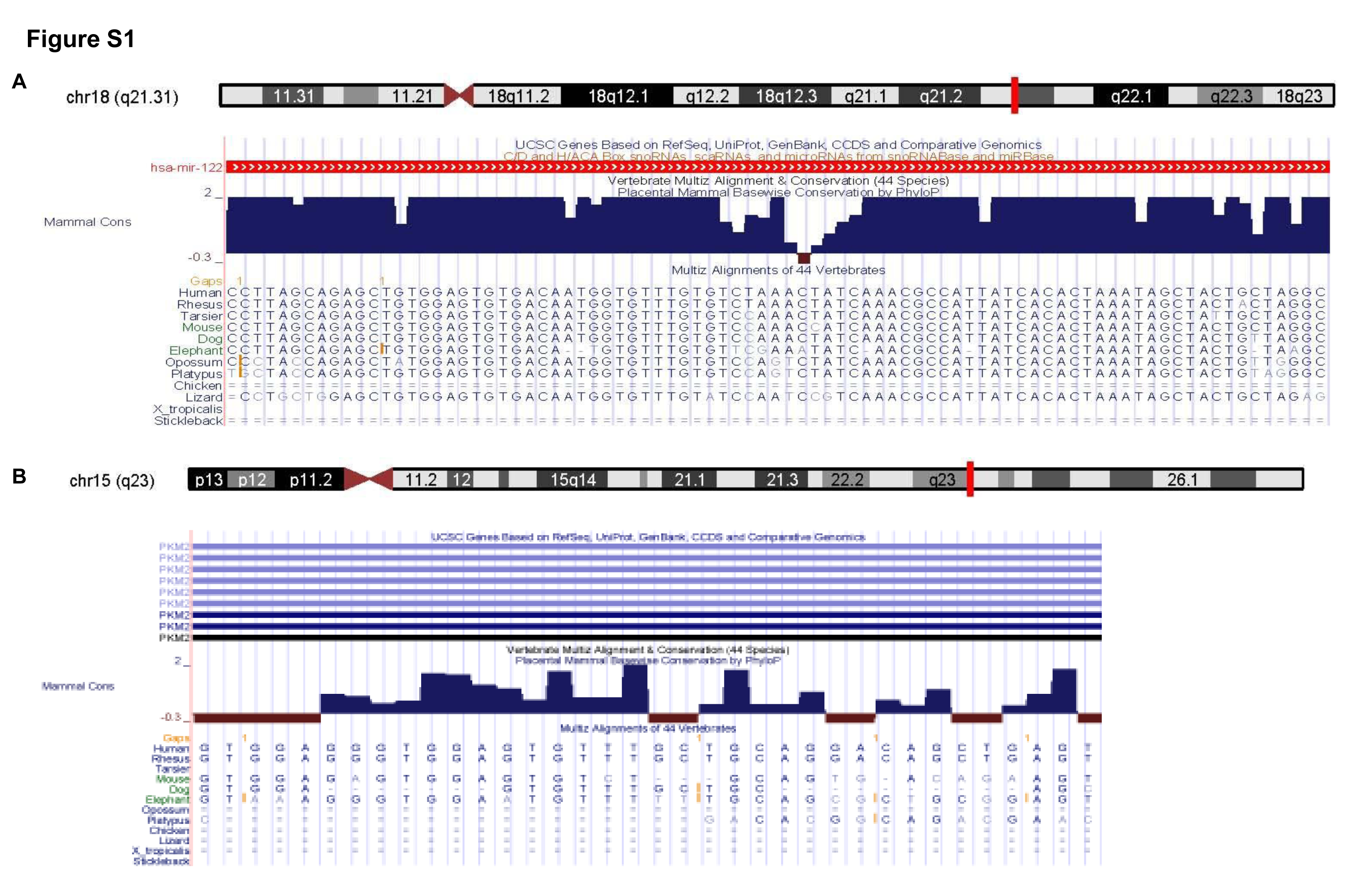

Supplement: Figure S1 — Genomic location and conservation of miR-122 and Pkm2 . (A) The location of miR-122 on chromosome 18 q arm is marked with a red bar. miR-122 is conserved in a large fraction of vertebrates. (B) The location of the Pkm2 3′UTR target sequence on chromosome 15 q arm is marked with a red bar. The target sequence predicted by MiRanda is located on 15:70,278,518–70,278,545 and the target sequence predicted by RNAhybrid is located on 15:70,278,510–70,278,528. Pkm2 is conserved in a large fraction of vertebrates. Data and images were generated using the UCSC Genome Browser (genome.ucsc.edu). (TIF) [file pone.0027740.s001.tif]

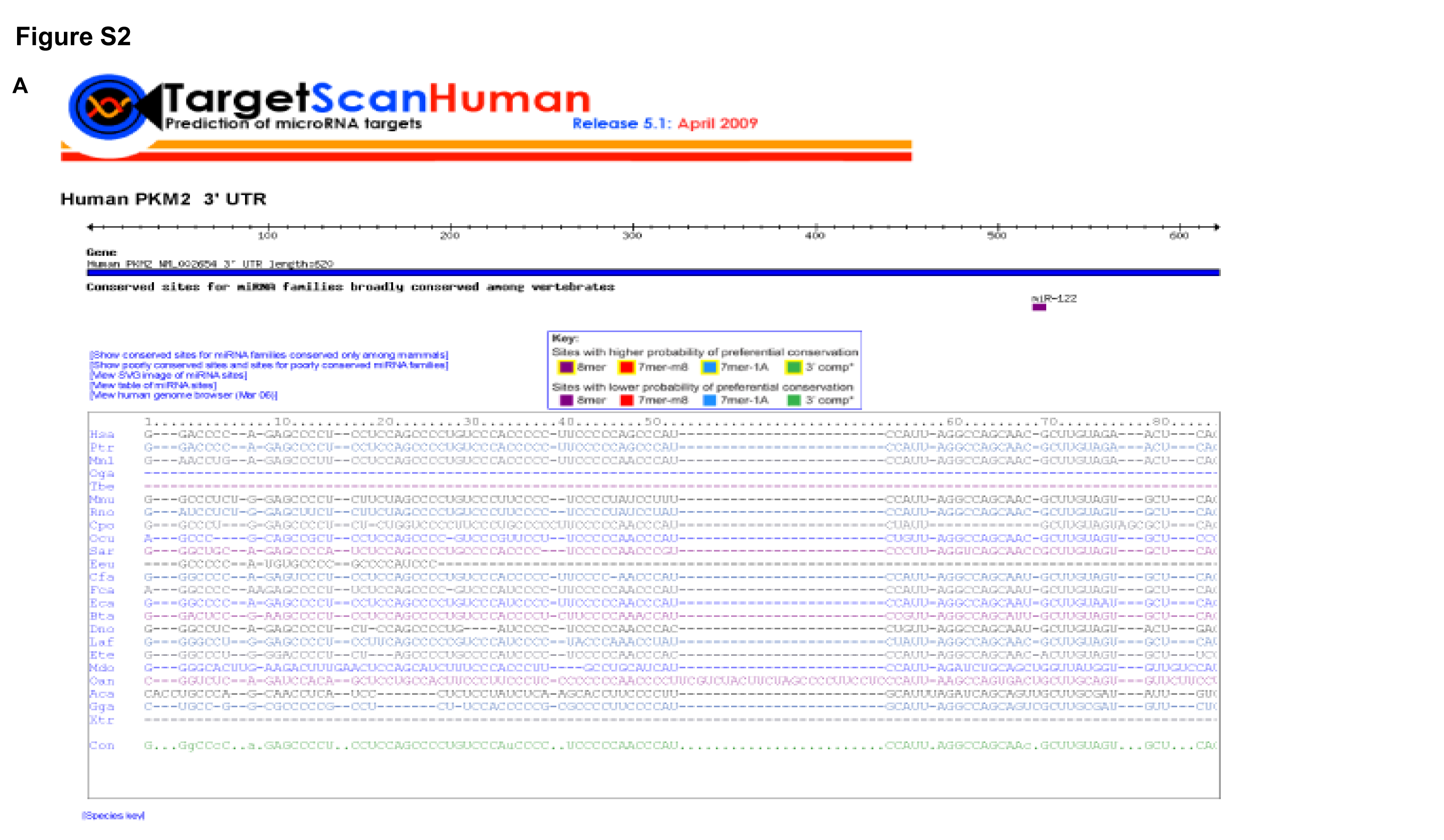

Supplement: Figure S2 — Genomic location and conservation of miR-122 and Pkm2 . (A) The location of Pkm2 is indicated by a blue bar, and the predicted hybridization site by miR-122 is indicated by the purple bar. The target sequence is predicted by Target Scan computational tool. The large boxed region shows that the target sequence of miR-122 in the 3′UTR of Pkm2 is conserved among a large number of species. Data and images were generated using the Target Scan computation prediction tool (www.targetscan.org). (TIF) [file pone.0027740.s002.tif]

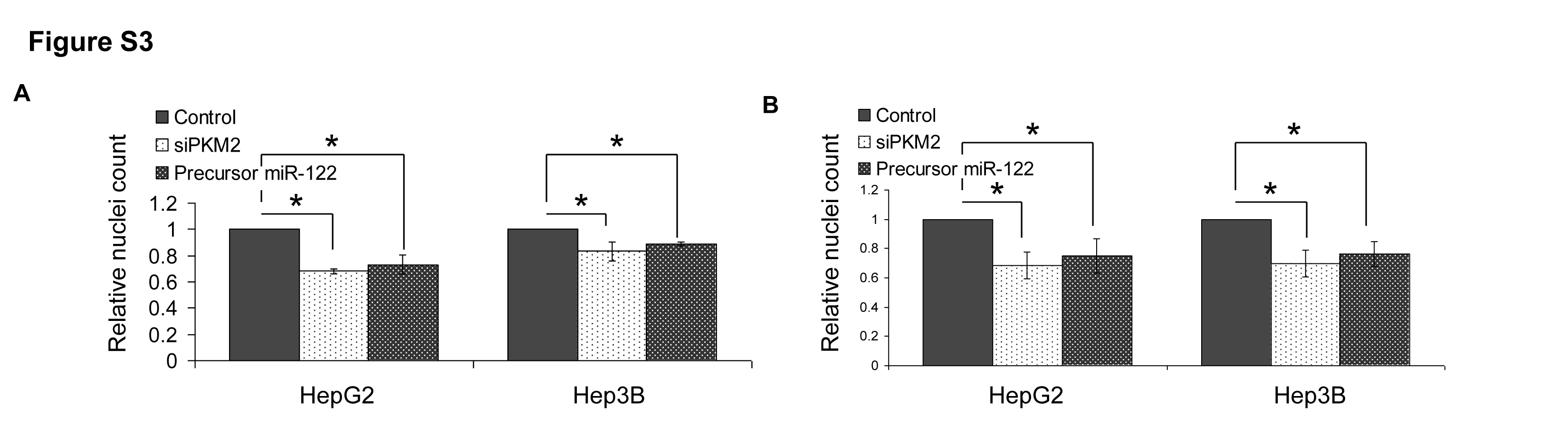

Supplement: Figure S3 — Reduction of endogenous Pkm2 expression by miR-122 modulates cellular proliferation. (A) HepG2 and Hep3B cells were transfected with siPkm2, precursor miR-122 molecules, or mock miRNA molecules. 24 hours (S1A) and 36 hours (S1B) post-transfection, five randomly chosen areas from 5 different wells in 24-well culture plates were stained with Hoecht and counted for the number of nuclei. n = 5; error bars represent s.e.m.; Student's t-test results are indicated by (*) P<0.05 and (**) P<0.01 relative to the control. (TIF) [file pone.0027740.s003.tif]

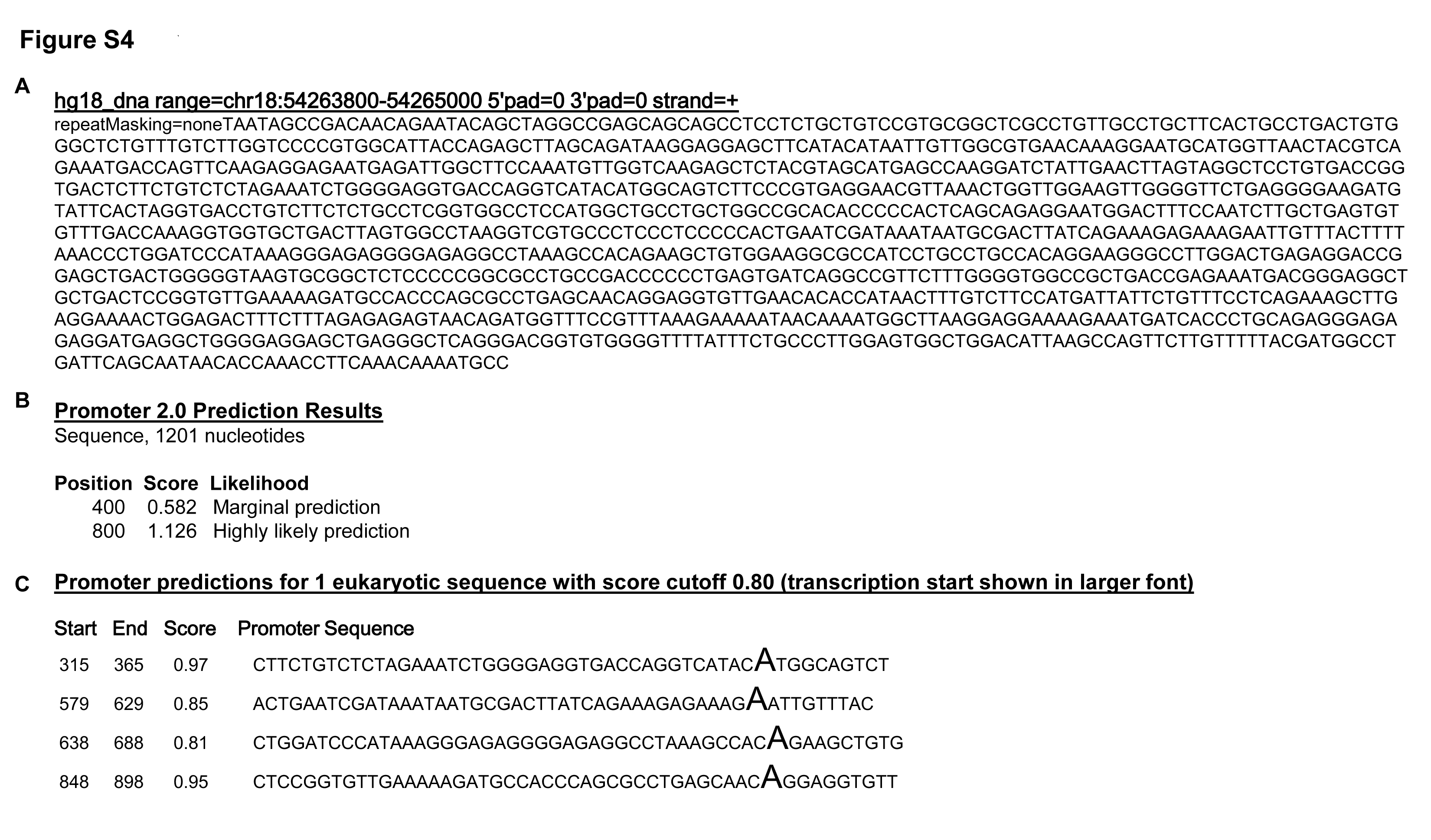

Supplement: Figure S4 — Predicted transcription start site of vertebrate RNAPII using Promoter2.0 and BDGP. (A) Genomic region between chr18:54,263,500–54,269,000 was evaluated for possible transcription start sequences using Promoter2.0 and Berkeley Drosophila Genome Project (BDGP). DNA sequence between chr18:54,263,800–54,265,000 yielded a highly likely promoter sequence (B) at position 800. (C) Predicted promoter sequences based on BDGP. Data and images were generated using Promoter 2.0 (cbs.dtu.dk) and BDGP (www.fruitfly.org). (TIF) [file pone.0027740.s004.tif]

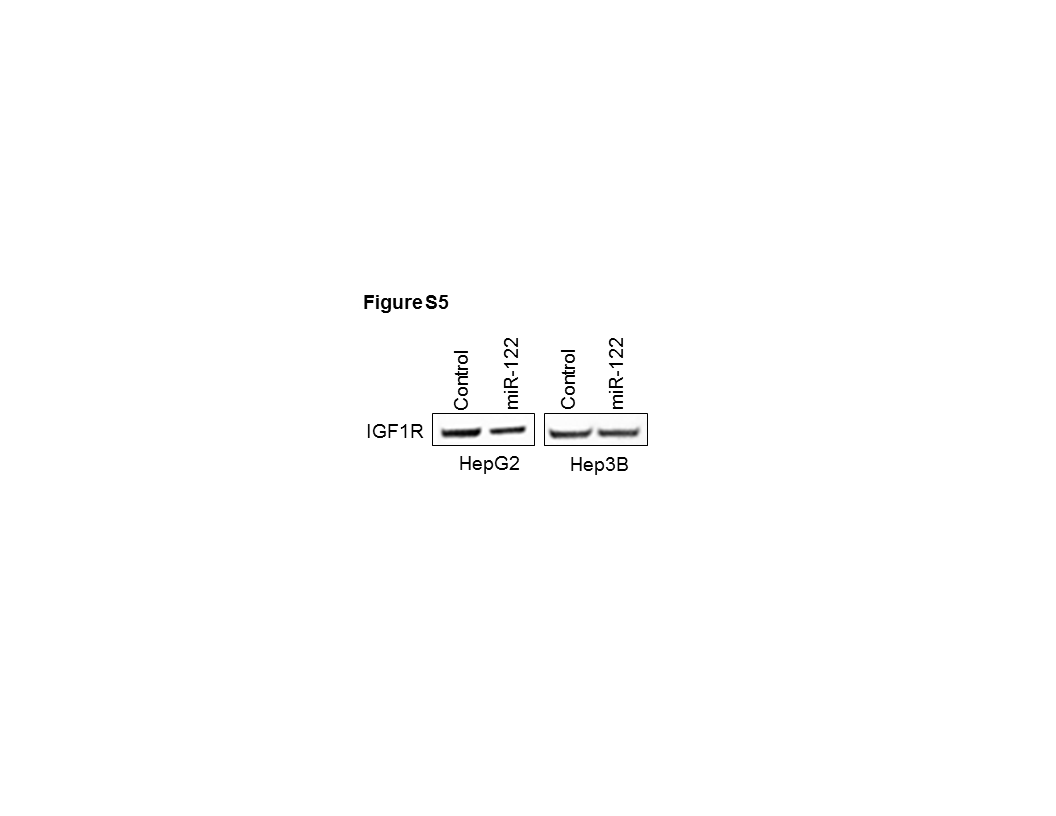

Supplement: Figure S5 — Overexpression of miR-122 is inversely correlated with IGF1R in HepG2 and Hep3B. Western blot assay of IGF1R in HepG2 and Hep3B overexpressing miR-122. (TIF) [file pone.0027740.s005.tif]
